# High-Resolution HLA-A Typing in Normal Iranian Population

**DOI:** 10.22034/ibj.22.2.134

**Published:** 2018-03

**Authors:** Azadeh Hadadianpour, Mohammad Hasan Samiee Aref, Sirous Zeinali

**Affiliations:** 1 Department of Biotechnology, College of Science, University of Tehran, Tehran, Iran; 2 Kawsar Human Genetics Research Center, Tehran, Iran; 3 Biotechnology Research Center, Pasteur Institute of Iran, Tehran, Iran

**Keywords:** HLA, Unrelated donors, Iran

## Abstract

**Background:**

Human leukocyte antigen (*HLA*) gene is a highly polymorphic region. HLA typing is required to match patients and donors for transplantation; therefore, development of HLA registries is necessary for finding HLA match donors. HLA system is highly informative, and numerous studies have been conducted on *HLA* allele distribution in different populations.

**Methods:**

In this study, 100 unrelated Iranian individuals were typed for HLA-A locus using sequence-based typing method. Samples were subjected to the PCR, followed by Sanger sequencing and software analysis.

**Results:**

A*02:01 (13%) and A*24:02 (12%) were the two most frequent alleles, while A*01:14, A*02:05, A*02:11, A*02:34, A*02:50, A*11:04, A*23:02, A*24:34, A*25:01, A*26:09, A*26:43, A*29:67, A*30:54, A*31:02, A*31:66, A*32:03, A*32:04, A*33:03, and A*66:15 alleles had the least frequencies (1%).

**Conclusion:**

This is the first report of HLA-A allele level typing in a randomized population of Iran and can be useful for development of national registries of HLA-typed volunteer marrow donors and local cord blood banks.

## INTRODUCTION

The major histocompatibility complex (MHC) is a fascinating genetic region encoding human leukocyte antigens (HLA)[1]. MHC molecules show an impressive degree of polymorphism that may have been resulted from selective pressures and functional adaptation during the evolution[1]. This high-degree polymorphism is essential for MHC molecule role in the regulation of immune system. Highly polymorphic MHC molecules can accommodate various antigenic peptides from invading pathogens and present these molecules to the T lymphocytes. Therefore, specific immune responses, unique to each person[2], are generated. HLA varieties among individuals are the major cause of organ and bone-marrow transplant rejection due to their ability to recognize self-from non-self-antigens[3]. The degree of donor and recipient HLA compatibility predicts the severity of graft versus host disease, the rate of transplant success, and the quality of patient’s survival[4]. High-resolution HLA typing at the allele level has significant functional implications for successful bone marrow trans-plantation and vaccine development[4,5]. Therefore, HLA typing has been used as an invaluable tool in population genetics analysis, studies of disease associations, and anthropological investigations[6]. HLA polymorphism in different populations and ethnic groups has been studied extensively, and results indicate that distinct populations have different allele frequencies and exhibit different patterns of linkage disequilibrium[7-10].

PCR-sequence-based typing (SBT) is a method generally used for obtaining high-resolution 4-digit allele level for HLA-A. This technology employs PCR to amplify the locus of interest. Sanger sequencing is subsequently used to determine the nucleotide sequence of the PCR products[11]. The present study was designed to investigate HLA class I allele frequencies for *HLA-A* gene in a randomized healthy population of Iran using the high-resolution SBT method. The results from this study can be useful for development of national registries of HLA-typed volunteer marrow donors and local cord blood banks.

## MATERIALS AND METHODS

### Sample

The present study includes 100 anonymous samples (50 male and 50 female) randomly collected from healthy unrelated individuals who referred to a private Medical Genetics Lab (Tehran, Iran) for genetic tests. These individuals did not suffer from any known disease and allergy, and they were not on any medications at the time. Participants were chosen based on the ethnic composition of Iran as follows: Persians (including Gilakis and Mazandaranis; n = 50), Baloch (n = 20), Azerbaijanis (n = 16), Kurds (n = 6), Lurs (including Bakhtiari people; n = 6), Arabs (n = 2). After *obtaining signed informed consents* from all the participants, the study was initiated.

### HLA-A allele level typing

Blood samples were collected in EDTA tubes. Genomic DNA was purified from whole venous blood using salting-out extraction method[12]. Quantification of DNA samples was conducted using the Thermo NanoDrop 2000 Spectrophotometer (Thermo Fisher Scientific, USA). High-quality DNA samples with the appropriate concentration of 20-30 ng/µL with the purity of A260/A280 around 1.6-1.8 were selected and were typed to four digits for HLA-A locus using commercial SBT Resolver™ Kit (Conexio-Illumina, USA) according to the manual’s instruction. The results were analyzed by Assign-SBT v4.7 software from Conexio-Illumina.

## RESULTS

In this study, 100 DNA samples from unrelated normal individuals were typed for HLA class I, using a reliable and an efficient *high-resolution* typing *method*. A total of 39 different alleles were detected for HLA-A locus in Iranian population. The most frequent allele groups were A*02, A*24, A*01, and A*03 with the frequency above 10%, and the least frequent allele groups were found to be A*66 and A*25 (1%). The complete list of alleles with their frequencies is shown in Table 1. The most frequent alleles were A*02:01 (13%) and A*24:02 (12%), followed by A*01:01 (9%) and A*03:01 (9%). However, the least frequent alleles were A*01:14, A*02:05, A*02:11, A*02:34, A*02:50, A*11:04, A*23:02, A*24:34, A*25:01, A*26:09, A*26:43, A*29:67, A*30:54, A*31:02, A*31:66, A*32:03, A*32:04, A*33:03, and A*66:15; all with the frequency of 1%. Most of the samples were heterozygote, while some alleles were observed as homozygous such as A*02:05:01, A*26.01, and A*01:01:01.

**Table 1:** HLA-A allele frequencies obtained in the Iranian population (n = 100)

HLA-A alleles	Allele frequencies (%)
A*01:01	9
A*01:02	2
A*01:14	1
A*02:01	13
A*02:02	2
A*02:05	1
A*02:11	1
A*02:34	1
A*02:50	1
A*03:01	9
A*03:02	2
A*11:01	5
A*11:04	1
A*23:01	2
A*23:02	1
A*24:02	12
A*24:03	2
A*24:34	1
A*25:01	1
A*26:01	5
A*26:09	1
A*26:43	1
A*29:01	2
A*29:67	1
A*30:01	5
A*30:54	1
A*31:01	4
A*31:02	1
A*31:66	1
A*32:01	4
A*32:03	1
A*32:04	1
A*33:01	3
A*33:03	1
A*66:15	1

## DISCUSSION

Numerous studies have focused on the distribution of HLA alleles in different populations due to its functional polymorphism. However, few studies are concerned with HLA allele frequencies in Iranian populations and mostly have drawn the attention to polymorphism in HLA class II loci[8,13] and to allelic association with diseases[14]. Most of the data presented in these reports were obtained by low-resolution HLA typing methods such as PCR-SSP[15,16]. Using molecular HLA typing technique, similar studies have reported A*02011 (20.2%) and A*11011 (14.6%) as the most frequent alleles in Baloch as well as A*24:02 (17.6%) and A*02:01 (12.5%) as the most frequent alleles in Kurd populations[17,18]. However, this is the first report of HLA-A allele level typing in a randomized Iranian population including different ethnicity groups.

The results indicated that the distribution of HLA-A alleles in Iranian population was mostly similar to Caucasians. For instance, the most common alleles found in Caucasians were A*02 (27%) and -A*24 (10.8%)[19]. Furthermore, HLA-A*02 (15.45-30.65%), -A*11 (16.66-30.72%), and -A*24 (11.03-17.07%) were the first three frequent alleles of HLA-A locus in six different Chinese populations[7]. Similarly, A*02 (29%) and A*24 (22.4%) had the highest frequencies in Korean population[10]. HLA-A*0201 and -A*0101 have also been reported as the most frequent alleles in a Berber population from North Morocco, named Metalsa[20]. Comparing to the HLA-A allele distribution in Armenian population, the most frequent HLA class I alleles were HLA-A*0201 (15.5%), -A*0101 (12.5%), and -A*2402 (12%), while HLA-A*25, -A*34, -A*66, and -A*74 were rare allele groups with a frequency of less than 1%. All of these findings support the genetic similarity between these neighboring and related countries[9].

Information provided by allele level HLA typing is of high importance for the bone marrow transplant community and vaccine studies. Furthermore, these kinds of research focusing on the allele and haplotype frequencies in Iranian population could be useful for finding the best matched unrelated donors in Iran.

The data of this study may be used as a reference by Iran’s hematopoietic stem cell donor bank and local registries to evaluate the immunogenetic profile of populations in specific regions and can impact investigations for unrelated bone marrow donors. However, further studies are required on other HLA loci and also on a larger sample size to achieve more precise results for development of HLA registries and cord blood banks.
